# Intraoperative 3D imaging in the treatment of elbow fractures - a retrospective analysis of indications, intraoperative revision rates, and implications in 36 cases

**DOI:** 10.1186/s12880-016-0126-z

**Published:** 2016-03-18

**Authors:** Marc Schnetzke, Julia Fuchs, Sven Y. Vetter, Nils Beisemann, Holger Keil, Paul-Alfred Grützner, Jochen Franke

**Affiliations:** Department for Trauma and Orthopaedic Surgery, MINTOS - Medical Imaging and Navigation in Trauma and Orthopaedic Surgery, BG Trauma Center Ludwigshafen at Heidelberg University Hospital, Ludwig-Guttmann-Strasse 13, 67071 Ludwigshafen am Rhein, Germany

**Keywords:** Elbow surgery, Intraoperative imaging, 2D fluoroscopy, 3D imaging, Revision, Complication

## Abstract

**Background:**

Three-dimensional (3D) imaging with a mobile C-arm has proven to be a valuable intraoperative tool in trauma surgery. However, little data is available concerning its use in the treatment of elbow fractures. The aim of the current study was to determine the intraoperative findings and consequences of 3D imaging in the treatment of elbow fractures.

**Methods:**

Between 2001 and 2015, prospectively collected data of 36 patients who underwent intraoperative 3D imaging during elbow surgery were recorded. The findings and consequences of the intraoperative 3D scans were analyzed in a retrospective chart review. For clinical evaluation the analysis included the patients’ medical history, the injury pattern of the affected elbow and concomitant injuries. Intraoperative and postoperative complications and revision surgeries were evaluated as well.

**Results:**

In 6 patients (16.7 %) analysis of the intraoperative 3D scan led to an immediate revision due to the detection of intra-articular screw placement (*n* = 3, 8.3 %) and remaining intra-articular step of >2 mm (*n* = 3, 8.3 %). In all of these patients, correct implant positioning and anatomical reduction could be achieved after immediate intraoperative revision, which was verified by a repeated intraoperative 3D scan. None of the 36 patients needed surgical revision based on postoperative radiological examinations due to secondary dislocation, wrong implant placement or remaining steps in the articular surface.

**Conclusions:**

Intraoperative 3D imaging offers additional information about fracture reduction and implant positioning in the treatment of elbow fractures compared to conventional intraoperative 2D imaging. It may therefore reduce the need for revision surgery. The value of intraoperative 3D imaging for clinical outcomes still needs to be assessed.

## Background

Three-dimensional (3D) imaging with a mobile C-arm has proven to be a valuable intraoperative tool in trauma surgery [1–5]. Since its first application at the start of the 21st century, intraoperative 3D imaging has become increasingly important in the treatment of displaced intra-articular fractures [5]. Several cadaveric and clinical studies have verified that diagnostic accuracy, fracture reduction and implant position can be improved by using intraoperative 3D imaging in different anatomical regions [6–8].

In complex anatomical conditions, where insight into the joint surface is not always possible or two-dimensional fluoroscopy may be unreliable due to overlapping structures, 3D imaging seems to be particularly helpful for the treatment of complex intra-articular fractures [5]. The main anatomical application areas for intraoperative 3D imaging are the calcaneus, spine, pelvis, tibial plateau, talus, ankle joint and wrist [5, 9–14].

Even though the elbow is easily assessable to intraoperative 3D imaging, only one report with three patients is available in the literature [1]. Intra-articular fractures of the elbow are prone to revision surgery in the case of secondary dislocation, intra-articular position of screws or remaining gaps after primary surgical treatment. In the treatment of elbow fractures a satisfactory outcome relies on anatomical reconstruction of the joint surface and proper implant placement [15–19]. Based on this background, intraoperative 3D imaging may be beneficial in the treatment of elbow fractures to improve outcome and prevent revision surgeries.

We therefore analyzed surgeries with treatment of elbow fractures where 3D imaging was used. The main aim of this study was to determine the intraoperative findings and consequences of 3D imaging in the treatment of elbow fractures. Secondarily, injury patterns around the elbow that could benefit from intraoperative 3D imaging, complications and revision surgeries were determined.

## Methods

Patient’s data with intraoperative 3D imaging of elbow fractures at a Level-I trauma center were analyzed in a retrospective chart review. All surgical procedures between September 2001 and June 2015 with intraoperative 3D imaging of the elbow (index operation) were evaluated.

### Study population

Inclusion criteria for the analysis were: 1) treatment of elbow fracture with the application of intraoperative 3D imaging (index operation), 2) complete documentation of the intraoperative findings of the 2D and 3D imaging by the surgeon, and 3) availability of patient’s full medical history, intraoperative 3D scans and postoperative course. Exclusion criterion was age under 18 years. All the study data were collected on the basis of a normal standardized clinical investigation. Evaluation of data was done retrospectively and anonymously. In agreement with the local ethics committee of the board of Medical Profession of Rhineland-Palatinate in Mainz this study did not require approval of the ethic committee.

### Imaging technique

**Fig. 1:**
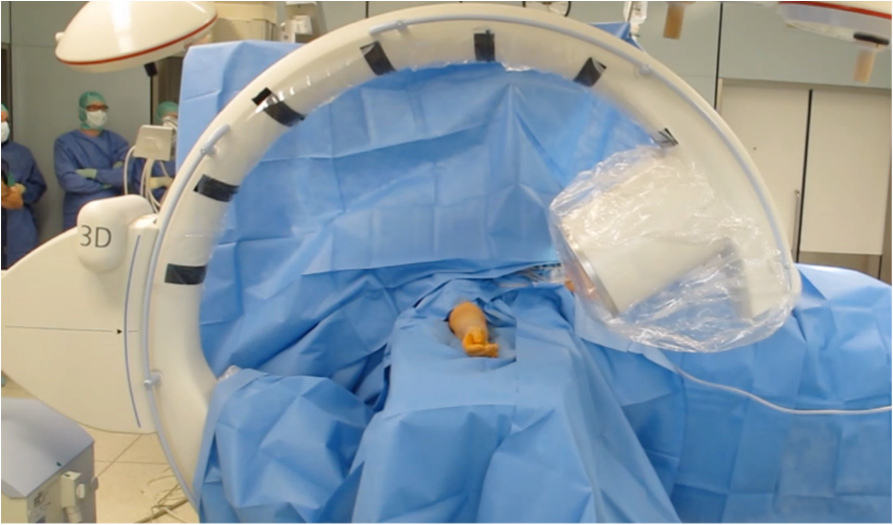
Intraoperative setting during the 3D scan with the elbow in the isocentre of the C-arm. The operating room personnel can stand outside the controlled area

**Fig. 2:**
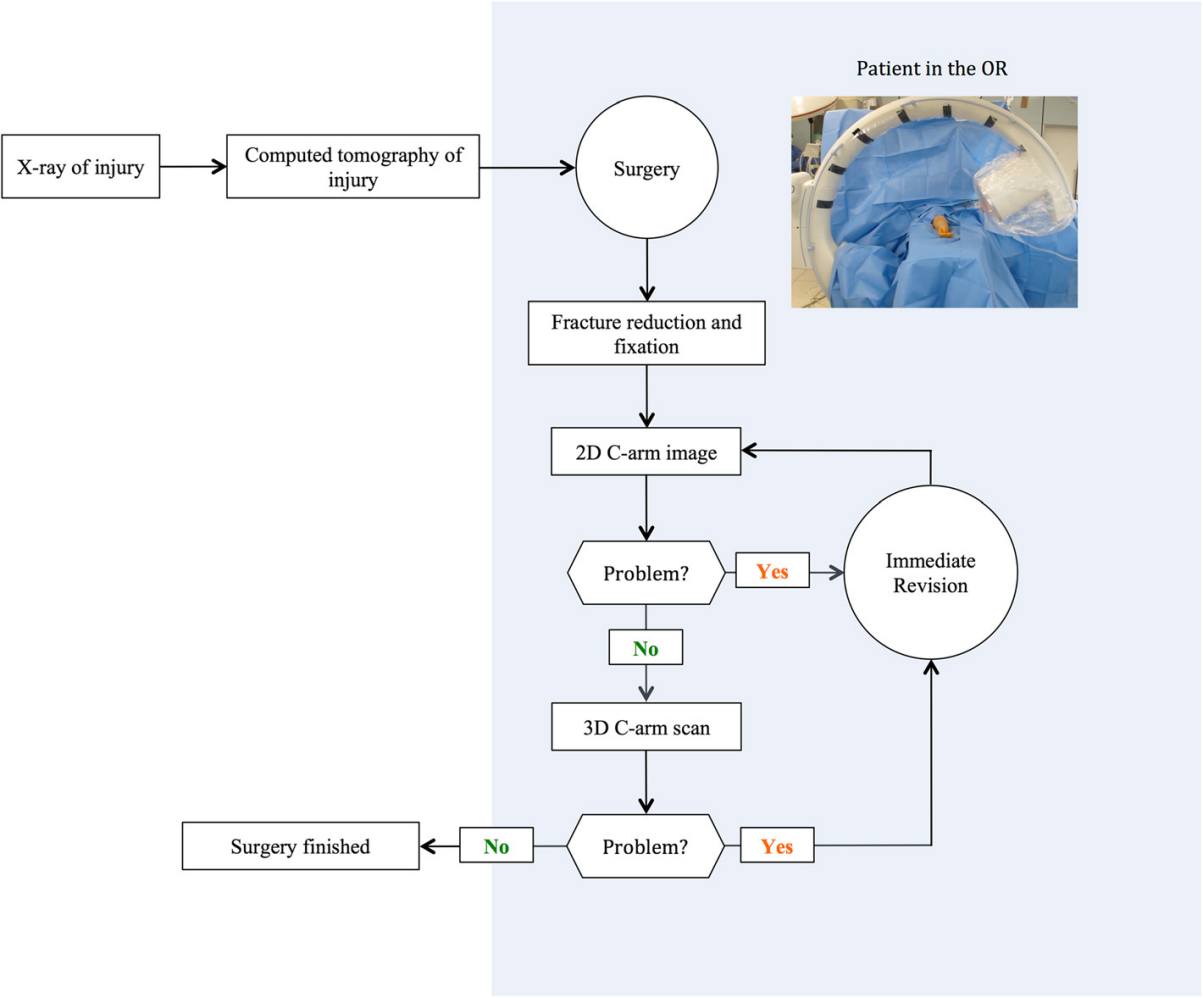
Flowchart of the workflow using the intraoperative 3D scan (OR: operating room)

In all patients the upper limb was positioned on a radiolucent carbon fiber arm-table for the operative procedure. The C-arm position is perpendicular to the upper limb. The intraoperative setting is illustrated in Fig. 1. After initial operative reduction and fixation, conventional 2D imaging with a mobile C-arm was performed using standard antero-posterior (AP) and lateral projections. The surgeon evaluated the images; if fracture reduction and implant position were judged to be appropriate, an intraoperative 3D scan was performed. In case of immediate intraoperative revision, the same sequence of intraoperative imaging was followed as before, i.e., fluoroscopy and then intraoperative 3D scan (Fig. 2).

The intraoperative 3D scan was performed using a SIREMOBIL Iso-C3D scanner (Siemens, Erlangen, Germany) early in the series. From March 15, 2005 onwards the ARCADIS Orbic 3D scanner (Siemens) was used and from 2011 onwards second-generation ARCADIS Orbic 3D was used. Detailed intraoperative findings, the number of 3D scans, and the consequences of each 3D scan were documented by the surgeon immediately after each surgery.

### Analysis of data

For each patient the medical history and previous surgeries of the affected elbow were assessed. The mechanism of injury, the injury pattern of the elbow and concomitant injuries were recorded. Fractures of the distal humerus were classified according to the Müller-AO Comprehensive Classification [20] by the operative surgeon. Additionally intra- and postoperative complications and revision surgeries were determined from the medical record.

All intraoperative 3D scans were analyzed for correct positioning of the implants and quality of fracture reconstruction. For this retrospective analysis, four possible evaluation criteria were used for the interpretation of the 3D scan: 1) correct implant positioning and anatomical reconstruction, no revision 2) correct implant positioning, no anatomical reduction achieved, no revision, 3) intra-articular screw-placement, immediate intraoperative revision, and 4) correct implant positioning, no anatomical reduction, immediate intraoperative revision.

### Statistics

Mean and standard deviation (SD) were calculated for continuous variables, and mean and median were calculated for ordinal variables. The Student’s *t*-test was used for statistical analysis. A two-tailed *p* value of <0.05 was considered to show a significant difference.

## Results

A total of 36 patients received 3D imaging of the elbow comprising 15 women (41.7 %) and 21 men (58.3 %). Mean age was 40.1 ± 18.9 years. In 19 patients (52.8 %) the right elbow was involved and in 17 patients (47.2 %) the left one.

### Medical history

In 26 patients (72.2 %) the injury was caused by a low energy trauma and in 10 patients (27.8 %) by a high-energy trauma. Ten patients (27.8 %) had concomitant injuries, 3 patients (8.3 %) were classified as polytrauma patients. At the time of injury, 29 patients (80.6 %) were healthy and 7 patients (19.4 %) had comorbidities such as hypertension (*n* = 3, 8.3 %) and atrial fibrillation (*n* = 2, 5.6 %). No patients had malignancies or metabolic disease.

### Injury pattern

Half of the patients in this study population had fractures of the distal humerus (*n* = 18, 50 %). In 7 of these patients (19.4 %) it was an isolated capitellum fracture. According to the AO all capitellum fractures were classified as B3 and the distal humerus fractures were classified as B1 (*n* = 3; 8.3 %), B2 (*n* = 4, 11.1 %), C1 (*n* = 3, 8.3 %) and C3 (*n* = 1, 2.8 %). None of the patients with a distal humerus fracture underwent an olecranon osteotomy. Table 1 shows the distribution of injury patterns for all patients.

**Table 1:** Distribution of injury pattern

Diagnosis	No. of patients (%)
distal humerus fracture	11 (30.6)
capitellum fracture	7 (19.4)
radial head fracture	6 (16.7)
olecranon fracture	6 (16.7)
elbow dislocation with intra-articular fracture	6 (16.7)

In 25 patients (69.4 %) intraoperative 3D imaging was used in primary treatment of the elbow fracture 6.7 ± 4.9 days after injury. In 8 patients (22.2 %) the index operation was performed due to implant failure or secondary dislocation of a previous surgery (Table 2). Mean time between failed primary surgery and index operation was 15.6 ± 15.5 days. In another 3 patients (8.3 %) an external fixator was applied during acute care surgery because of soft tissue swelling. Mean duration between application of the external fixator and index operation was 19.7 ± 21.1 days, in one of these patient the definite treatment could only be done 44 days after injury.

**Table 2:** Reasons for the index operation after failed primary treatment

Reasons leading to index operation	No. of patients (%)	Time between primary treatment and index operation (d ± SD)
Secondary dislocation	5 (13.9)	15.6 ± 15.5
Incorrect reposition	2 (5.6)	11 ± 9.9
Chronic instability	1 (2.8)	100

### Analysis of intraoperative 3D scans

In 36 index operations a total a 45 intraoperative 3D scans were performed. In 30 patients (83.3 %) a single 3D scan was performed, in 5 patients (13.9 %) the 3D scan was done twice and in one patient (2.8 %) the scan was repeated 4 times until anatomical reduction and correct implant positioning were achieved. Analysis of the data showed that an average of 1.9 ± 1.8 elbow surgeries with one ore more intraoperative 3D scans per year were performed between September 2001 and December 2013. In 2014 and 2015, the number of elbow surgeries with application of intraoperative 3D scans increased to 6.0 ± 4.2 per year. This difference was statistically significant (*p* = 0.024).

The application of intraoperative 3D imaging revealed pathological findings in 12 patients (33.3 %), which was not seen on conventional 2D fluoroscopy (Table 3). In 6 patients (16.7 %) the 3D scan led to an immediate revision due to the detection of intra-articular screw placement (*n* = 3, 8.3 %) or remaining intra-articular step (*n* = 3, 8.3 %) (Table 4).

**Table 3:** Analysis of the findings of intraoperative 3D imaging

Findings in 3D imaging	Visible on 2D fluoroscopy	Immediate revision	No. of patients (%)
correct implant positioning and anatomical reconstruction	Yes	No	22 (61.1)
correct implant positioning, remaining step <2 mm	No	No	6 (16.7)
intra-articular screw-placement	No	Yes	3 (8.3)
correct implant positioning, remaining step >2 mm	No	Yes	3 (8.3)

**Table 4:** Patients with intraoperative revision due to findings of the intraoperative 3D scan

Patient No.	Injury	3D findings	Consequence of 3D scan	No. of 3D scans
1	Olecranon fracture	Intra-articular screw placement	Screw replacement	2
2	Capitellum humeri fracture 13B3	Intra-articular screw placement	Screw replacement	2
3	Distal humerus fracture 13B1	Remaining step >2 mm	Improvement of reconstruction	5
4	Fracture of the coronoid process type III (Regan & Morrey)	Remaining step >2 mm with persistent instability	Improvement of reconstruction	2
5	Distal humerus fracture 13C3	Remaining step >2 mm	Improvement of reconstruction	2
6	Distal humerus fracture 13C2	Intra-articular screw placement	Screw replacement	2

In all of these 6 patients, a correct implant positioning and satisfactory reconstruction could be achieved after immediate correction, which was verified by repeated intraoperative 3D scan.

In two patients (5.6 %) the intraoperative 3D scan was performed for diagnostic reasons. In one patient (2.8 %) the surgeon suspected an iatrogenic fracture of the distal humerus due to a brisement force of the elbow, which could not be certainly excluded on 2D fluoroscopy. With the application of 3D fluoroscopy, the suspicion of an iatrogenic fracture was definitely eliminated. A comparable case was recorded with a patient who had a chain injury of the upper limb and intraoperative suspicion of a radial head fracture, which was also not verified under 3D fluoroscopy.

### Postoperative complications and revisions

Four patients (11.1 %) developed a total of three early complications after the index operation (postoperative hematoma, superficial wound infection and palsy of the radial nerve) and one late complication (postoperative arthrofibrosis of the elbow) leading to three revision surgeries (removal of the hematoma, wound debridement and implant removal with open arthrolysis). The nerve palsy resolved completely under conservative treatment. Mean time between index operation and revision was 14 ± 2.8 days for the early complications and 135 days for the late complication. None of the revisions were related due to incorrect implant positioning or inadequate reconstruction of the joint surface.

### Examples for intraoperative 3D imaging

**Fig. 3:**
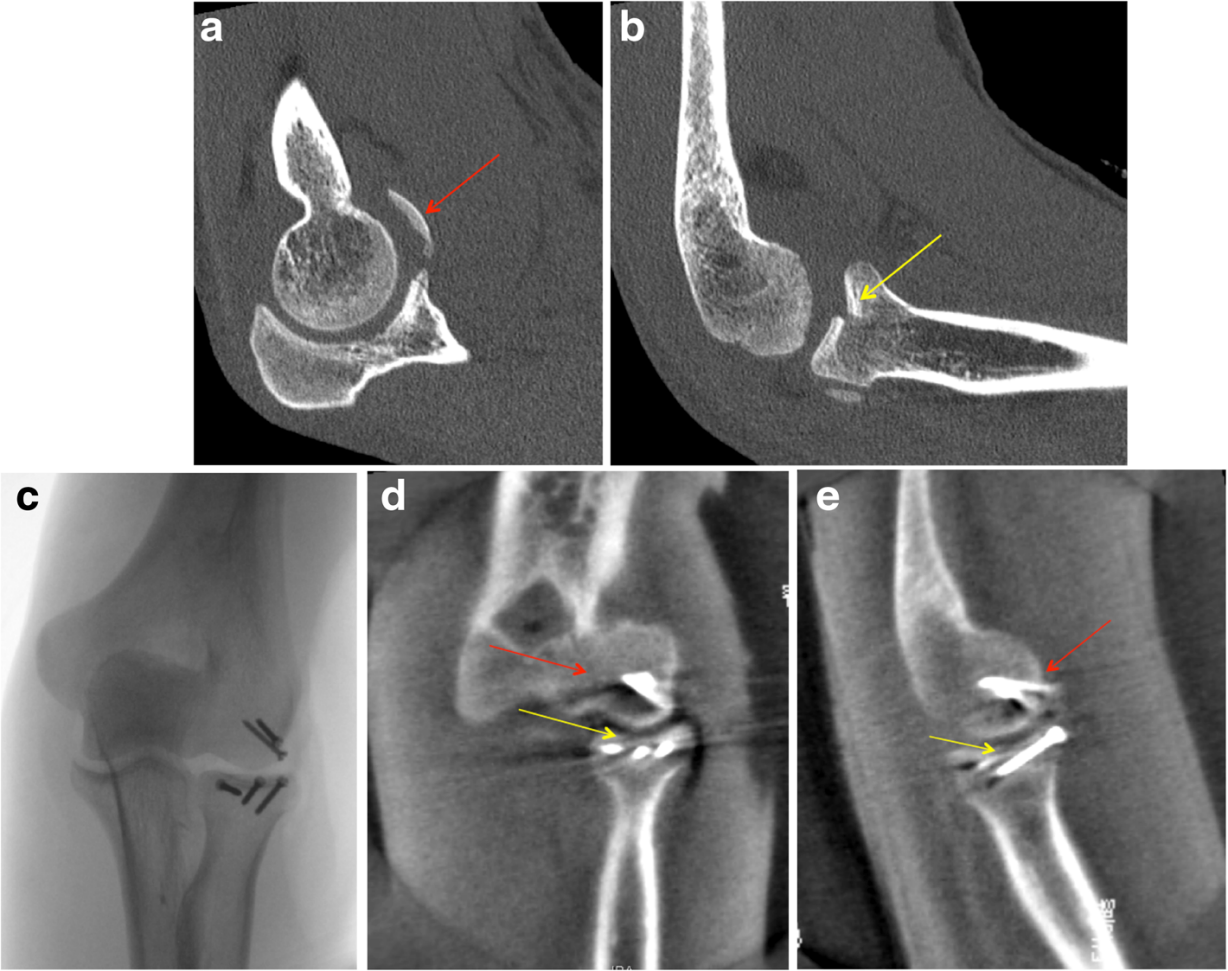
**a**, **b** Pre-operative computed tomography showing a complex elbow injury with a flake fracture (2x3cm, *red arrow*) of the capitellum and a radial head fracture type II according to Mason with a step of >2 mm in the joint surface (*yellow arrow*). **c** Intraoperative 2D fluoroscopy images after open reduction and fracture fixation with screws. **d**, **e** The fracture reduction of the capitellum could not be visualized intraoperatively. The intraoperative sagittal and coronal multi-planar-reconstructions of the 3D scan confirmed anatomical reduction of the capitellum (*red arrow*) as well as the radial head fracture (*yellow arrow*)

Patient No. 1 (Fig. 3a-e).

**Fig. 4:**
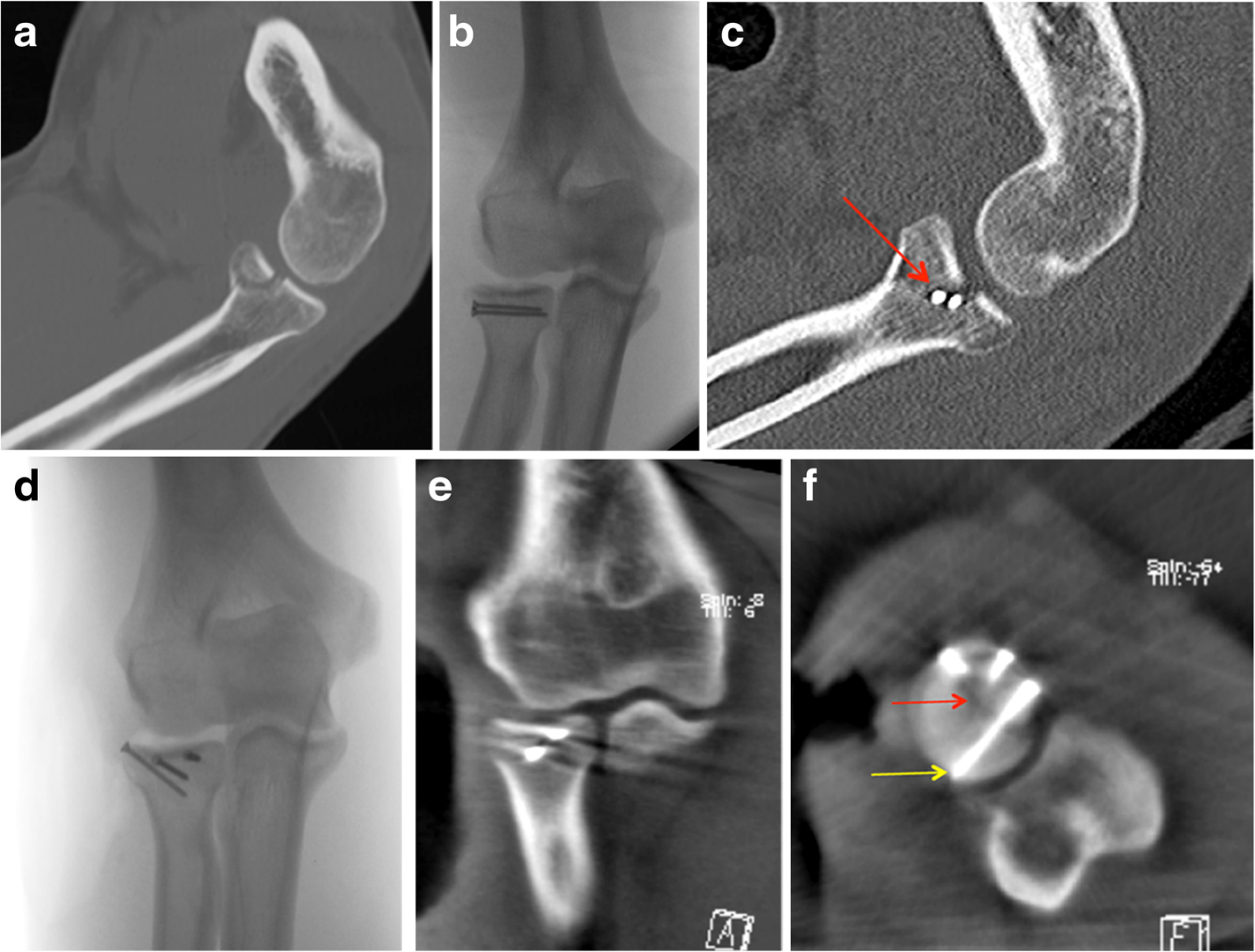
**a** Pre-operative computed tomography showing a radial head fracture type II (Mason). **b** Intraoperative 2D fluoroscopy images after open reduction and fracture fixation with screws showing anatomic reduction. **c** Postoperative computed tomography revealed a remaining step in the joint surface. Both screws were placed into the fracture line, which was not seen on conventional 2D fluoroscopy. **d** During an index operation, the screws were replaced after open reduction. **e** Intraoperative 3D imaging confirmed the anatomical reduction and correct screw positioning in the coronal multi-planar-reconstructions. **f** Intraoperative 3D imaging confirmed the anatomical fracture reduction (*red arrow*) and correct screw positioning und length (*yellow arrow*) in the axial multi-planar-reconstructions

Patient No.2 (Fig. 4a-f).

## Discussion

The aim of this study was to determine the intraoperative findings and consequences of 3D imaging in complex elbow surgery. Secondarily, complications and revision surgeries were reported as well.

In the present study intraoperative 3D imaging revealed pathological findings in 12 patients (33.3 %), which was not seen on conventional 2D fluoroscopy. In 6 patients (16.7 %) the intraoperative 3D scan led to an immediate revision due to the detection of intra-articular screw placement (*n* = 3, 8.3 %) or remaining intra-articular step of >2 mm (*n* = 3, 8.3 %). In all of these patients, a correct implant positioning and anatomical reconstruction could be achieved by immediate adjustment of implant placement and fracture reconstruction, which was then verified by repeated intraoperative 3D scan. The results of the current study support the notion that intraoperative 3D imaging in the treatment of elbow fractures is particularly helpful, when the fracture reduction and the joint surface cannot be completely visualized in the open operation situs or by conventional 2D fluoroscopy.

The surgical revision rate in elbow surgery is quite high compared to other anatomical regions [15–18], which is supported by the findings in this study. In 8 of 36 patients (22.2 %) the index operation was performed due to implant failure or secondary dislocation of a previous surgery. After the index operation, none of the 36 patients needed surgical revision based on postoperative radiological examinations for reasons of secondary dislocation, wrong implant positioning or remaining steps in the articular surface. So our study indicates that the use of intraoperative 3D imaging can prevent secondary revision surgery.

For distal humerus fractures (4 of 6 intraoperative revisions in this study) in particular, 3D imaging seems to be beneficial. Detailed analysis of patients with fracture localization at the distal humerus (*n* = 18, 50 %) showed that an olecranon osteotomy was not performed. The distribution of injury severity revealed that the distal humerus fractures in this study group were considerably complex according to the AO classification (14 type B, 4 type C). These findings might indicate, that the availability of an intraoperative 3D scan improves the possibility to treat intra-articular fracture of the distal humerus without an olecranon osteotomy. This could decrease the surgery time, the rate of complications related to the osteotomy, material costs, and the needs for implant removal [21, 22].

The relative number of patients who underwent an intraoperative 3D scan of the elbow is quite low. In other anatomical areas than the elbow application of intraoperative 3D imaging is much more frequent. In the treatment of ankle or calcaneal fracture we have learned that despite the fact, that the fracture appeared to be adequately reduced with correct implant placement on fluoroscopy, the intraoperative 3D scan often reveals findings, that need to be revised [11, 23]. These observations led to a higher application of intraoperative 3D imaging in the treatment of elbow fracture in 2014 and 2015 compared to the period from 2001 to 2013.

Comparing our results with the literature is difficult due to a lack of published data concerning 3D imaging and elbow surgery. There is one report of three patients with intraoperative 3D imaging by Carelsen et al. However this lacks a detailed analysis [1].

In other anatomical regions, the application of intraoperative 3D imaging has proven to be valuable. In the literature immediate intraoperative revision rates of 11 to 40 % are reported following application of intraoperative 3D imaging in complex intra-articular fractures [2–4, 11, 23–25]. The intraoperative revision rates of the current study (16.7 %) fall within this range. For the upper extremities there are very few reports about the application of intraoperative 3D imaging in fracture surgery. Carelsen et al. recently published their experiences with the use of intraoperative 3D imaging for wrist surgery and found that none of the 56 patients treated with the aid of 3D fluoroscopy required revision surgery [9]. The intraoperative revision rate was 10.5 %. Mehling et al. in reported in 2013 about 51 patients treated for distal radius fractures with the aid of intraoperative 3D scan. The authors compared the findings on conventional 2D fluoroscopy with intraoperative 3D scan and found, that in 31.3 % of the operations there were 17 malpositioned screws not shown on standard 2D fluoroscopy that were detected using intraoperative CT imaging [26].

This is in agreement with our finding and supports the idea that intraoperative 3D imaging provides the trauma surgeon with extra information that is not available from conventional fluoroscopy leading to a decrease in the rate of revision surgery. At present, no data are available in the literature about indications, intraoperative revision rate, and implications of 3D imaging in the treatment of elbow fractures. Our study is the first report about a reasonably large series of patients receiving intraoperative 3D imaging of the elbow.

A potential drawback for the use of intraoperative 3D imaging might be the higher radiation dose and the processing time of the 3D scan. The specific radiation dose was not measured in the current study. A previous study on wrist surgery reported, that intraoperative 3D imaging raised the dose-area product by 3.2 cGycm^2^ and increased the overall dose by 55.6 % on average [26]. The radiation exposure for the surgeons and the personnel staff, who are exposed to radiation almost every day, is practically reduced to zero because the 3D scan can be started from outside the controlled area (Fig. 1). Mehling et al. concluded in their study that the investment of about 3.2 cGycm^2^ and an extension of operation time of about 5 minutes enables the surgeon to minimize the need for revision surgery together with multiple radiation doses. Application of intraoperative 3D imaging with a mobile C-arm takes about 60 s and the whole process, preparation for the 3D scan, application, processing and analysis of the images take approximately 5 min. A cost analysis of intraoperative 3D imaging by Hüfner et al. has demonstrated that an economic benefit can be achieved if the revision rate is decreased by just 5 % [27]. In the current study none of the patients required postoperative conventional computed tomography or revision surgery due to implant failure or secondary dislocation.

This study has several limitations. The selection of the study population was based on the surgeon’s opinion during operative treatment. There was no randomization or control group. Furthermore, clinical outcome was not assessed and so no information can be provided for functional and radiological outcome. Even though all cases were included in a prospective database, evaluation of the data was retrospective and a power analysis was not performed.

## Conclusions

Intraoperative 3D imaging provides additional information for the treatment of elbow fractures that is not available from conventional methods such as physical examination and 2D fluoroscopy. Intraoperative 3D imaging seems to be particularly helpful when the fracture reduction and the joint surface cannot be completely visualized, e.g. in distal humerus fractures type B and C according to the AO classification. The availability of an intraoperative 3D scan might improve options to treat intra-articular fracture of the distal humerus without the need for olecranon osteotomy. That would decrease surgical time, complication rates and the need for implant removal.

Although 3D imaging has not evolved to become a routine procedure in the treatment of elbow fractures, it may reduce the need for revision surgery in complex intra-articular fractures. The value of intraoperative 3D imaging on functional outcomes still needs to be assessed.

### Data availability

Data are available on request from the corresponding author.

## Abbreviations

3D: three-dimensional
AP: antero-posterior
OR: operating room
SD: standard deviation
